# Health and Rehabilitation Science specialities, physical activity and dimensions of wellness among the students of PNU

**DOI:** 10.1016/j.heliyon.2020.e03204

**Published:** 2020-01-15

**Authors:** Uzma Zaidi

**Affiliations:** Department of Health Sciences, College of Health and Rehabilitation Sciences, Princess Nourah bint Abdulrahman University, Riyadh, Saudi Arabia

**Keywords:** Psychology, Wellness, Physical activity, Health, Rehabilitation, Students, Saudi Arabia

## Abstract

**Introduction:**

The dimensions of wellness and engagement in physical activity both are crucial for a healthy lifestyle, adjustment, and attainment of professional excel among young female health students.

**Objective:**

The purpose of the study is to determine adherence to physical activity and dimensions of wellness among students of the College of Health and Rehabilitation Sciences, Princess Nourah bint Abdulrahman University (n = 198).

**Design:**

This study is based on a Cross-sectional design.

**Setting:**

Students of College of Health and Rehabilitation Sciences, Princess Nourah bint Abdulrahman University in Riyadh, Saudi Arabia. The study was carried out from May to December, 2018.

**Material & methods:**

Perceived Wellness scale was used to measure six dimensions of wellness. The frequency of physical activity was measured by self-report 5-point Likert scale. Data was collected through survey research forms.

**Results:**

Results revealed that the physical wellness dimension was significantly correlated with all physical activity, exercising last year, social, intellectual, and overall wellness (p < .01) and with psychological, emotional, and spiritual wellness (p < .05). Furthermore, regression analysis confirmed the predictive relationship of frequency of physical activity and exercising last year (R^2^ = .113, F (2, 195) = 12.371, p < .01) with perceived wellness. The department-wise comparison of wellness perception revealed that all the departments score differently on various wellness dimensions.

**Conclusion:**

Comprehensively, association with all dimensions of wellness and physical activity was found among students of the College of Health and Rehabilitation. It further validates the impact of their well-designed curriculum and encouragement to engage with a healthy lifestyle. Further programs can work out to dimensions of wellness where weakness was found to achieve overall wellness.

## 1 Introduction

Some authors previously defined health as the wellness of the body, mind, and spirit (Rehman et al., 2013). Health professionals are individuals who ideally are in a position to promote health and wellness in their patients. According to the American Physical Therapy Association, a health professional needs to be competent in the ability to identify and instruct patients in the basic health promotion activities such as personal hygiene, exercises, and to engage in other health advocacy practices (American Physical Therapy Association (APTA), 2014). It implies that health students need to have general medical knowledge and skills. In the current scenario of Kingdom of Saudi Arabia (KSA), where women have just started enjoying an equal share in the progress of every field of life, it is necessary to explore the perception of wellness of female in general and health students, in particular, to involve them in community health and beyond. Moreover, the reported prevalence of inactivity in KSA made the issue more worth studying (Khateeb et al., 2019; Abdel-Salam and Abdel-Khalek, 2016).

Wellness encompasses the complete health of the individual (Kramer, 2015). Wellness has several dimensions, namely, physical, social, intellectual, spiritual, psychological, and emotional wellness (Stoewen, 2017). Physical wellness involves a variety of healthy behaviours and practices such as proper dietary behaviours, adequate exercises, and abstaining from harmful habits such as drug and substance abuse. Adhering to these practices and behaviours will guarantee an individual a healthy and better quality of life (Verhagen and Engbers, 2009). For optimal physical wellness, an individual ought to know how to identify illness symptoms, go for frequent medical check-ups, to maintain proper dietary practices, and to refraining from drug abuse (Fatima et al., 2013). Physical wellness eventually results in psychological, social, emotional, spiritual, and intellectual benefits (Rehman et al., 2013).

Social wellness makes individuals have successful interaction, communication, relationship and to feel appreciated and belonging (Kramer, 2015). Health professionals have suggested that for optimal social wellness, an individual need to cultivate healthy relationships, contribute to the community, share their skills and talents, and freely communicate their ideas, feelings, and thoughts (Rehman et al., 2013). Intellectual wellness demands that an individual uses the available resources to expand their knowledge and skills with an active mind (Midtgaard et al., 2008). Keeping with up to date events that arouse the mind is also critical (Sohail, 2013). Other ways to keep our intellectual wellness involves reading, learning, or perfecting on a foreign language, engaging with people who challenge one's intellect.

Spiritual dimension involves an individual having a set of guiding beliefs that help to direct one's life. It involves having an unwavering faith and unending commitment in ones' belief that provide a sense of meaning and purpose in life (Stoewen, 2017). Psychological wellness is firmly attached to emotional wellness. Emotional disturbances and trauma can lead to psychological issues, for instance, depression, anxiety, obsessions, and more severe ones like schizophrenia. Persistence of emotional disturbance creates a problem to approach psychological support and treatment (Center for Substance Abuse Treatment (US), 2014).

Health professionals have found that physical activity can improve immensely on health and reduce the risk of several diseases (Verhagen and Engbers, 2009). There are numerous benefits of physical activities, for instance, reducing the risk of heart attack, better weight management, lower blood cholesterol level, lower blood pressure level, stronger bones, muscles, and joints and feeling better with more energy and better mood (Centers for Disease Control and Prevention, 2015). Physical exercises help to reduce depression. With increased fitness, the mood is uplifted and improve the sleep pattern (Centers for Disease Control and Prevention, 2015).

The World Health Organization defined wellness as the optimal state of health of individuals and groups (Smith et al., 2006). Cohesively, wellness is an active and unique process of change that help an individual to reach their maximum potential, and that includes emotional, spiritual, intellectual, financial, physical, and social wellness. Thus, it clear that physical activity is a component that contributes to the wellness process (Fatima et al., 2013). Therefore, the physical components of physical wellness, including proper nutrition, adhering to routine exercises, all contribute to the wellness of an individual.

This study is based upon the Social cognitive theory that emphasises that behaviour can be learned by social interaction while living in a particular situation (Bandura, 1986). Furthermore, biopsychosocial circumstances are considered to impact the lifestyle of individuals substantially (Melchert, 2011). It becomes more potent among youth as they get easily influences by their surroundings. According to the Health Promotion Model (HPM), people adopt health-enhancing behaviours due to the biopsychosocial impact (Khodaveisi et al., 2017). It can be concluded that our actions are based on our beliefs (Khodaveisi et al., 2017).

The medical/health science students, future doctors, and therapists can not underestimate their importance about wellness (Paro et al., 2010). They have an obligation as future practitioners to expand their knowledge on wellness to help them better serve the patients. They ought to prescribe the suitable exercise practices to the patients, therefore, promoting physical activity awareness (Fatima et al., 2013). The medical students were found to have a high level of physical activity because of their university modules involving sports (Mikolajczyk et al., 2008). However, the degree of physical activity is different depending on different sports activities and diversity in culture. One of the studies from Jazan, KSA (Kahlafalla et al., 2017) reported that female medical students were less active compared to male students due to the unavailability of enough physical activity facilities.

As far as research evidence from Saudi Arabia is concerned related to physical activity among Saudi female medical students, some evidence was found in the literature. One of the studies based on BMI and dietary habits of Saudi female (Khalaf et al., 2015), another was related to physical activity engagement (Al-Bannay et al., 2017). Some other studies were presenting health and non-health students’ comparisons (Almutairi et al., 2018), but there was no evidence found for the relationship between physical activity and wellness perception. One of the recently published systematic review studies has presented reasons for physical inactivity among Saudi female was found correlated with lack of time, exercise facilities, and resource (Al-Hazzaa, 2018). Our actions are based on our beliefs (Khodaveisi et al., 2017). Therefore, the current study will try to fill the gap to investigate the relationship of physical activity with perceived wellness dimensions among final year female health students of Princess Nourah bint Abdulrahman University (PNU). PNU is a women-only University located in the metropolitan city of Riyadh; could facilitate to approach the female student population in one place to understand their physical activities and wellness perceptions. Moreover, the College of Health and Rehabilitation Sciences (CHRS) offers a wide range of programs; thus, it has excellent potential to understand the phenomenon of how discipline could cultivate healthy behaviour and perception of wellness among female Health students. It was hypothesized that:

Hypothesis 1 Physical activity will be associated with perceived wellness among students of Health and Rehabilitation Sciences.

Hypothesis 2 Department wise students of Health and Rehabilitation Sciences will score significantly different on dimensions of wellness (psychological, emotional, social, physical, spiritual, intellectual and overall wellness).

## 2 Methods

Research design: The present study used a cross-sectional, descriptive, correlational research design. The research method applied is quantitative. Survey technique was used, including standardised self-report questionnaires, rating scale, and demographic information sheets. The study was conducted to investigate the relationship between physical activity and perception of wellness among students of CHRS, PNU, in Riyadh during May–December, 2018.

Sampling: PNU has five (5) colleges in Health Campus. College of Health and Rehabilitation CHRS is the biggest and comprises a variety of programs and consists of four (4) departments, including Radiological Science, Rehabilitation Sciences, Health Sciences, and Communication Sciences. Furthermore, there are 13 program divisions within four departments (Princess Nourah Bint Abdurahman university (PNU), 2018). The sample of this study comprised of (n = 198) female students enrolled in BS programs of final year (level 7 & 8) at CHRS, PNU. Few studies were found related to stress in medical students (Abdulghani et al., 2011). BMI and physical activity (Khalaf et al., 2015), physical activity and health belief (Al-Bannay et al., 2017), and engagement in physical activity (Almutairi et al., 2018). One of the recent studies conducted in Jeddah for health professionals was considered to calculate size effect (Khateeb et al., 2019). Sampling formula for descriptive study was utilized by conducting openepi application (Sample size *n* = [DEFF*Np(1-p)]/[(d^2^/Z^2^_1-α/2_*(N-1)+p*(1-p)]) (Charan and Biswas, 2013). Total population (N) was 255 students enrolled in the final year of CHRS, an expected proportion of the population (p) with regular physical activity was 75%. The margin of error (d) was 5%, and the design effect for the cluster survey was determined as 1.5. A total sample size of 204 students was computed. The further sample size was divided into four departments including Rehabilitation sciences (80/255*204 = 64), Health Sciences (94/255*204 = 75.2), Radiological sciences (39/255*204 = 31.2), and communication sciences (42/255*204 = 31.6). At the third level of sample division proportionally, a calculation was done for 11 educational programs respectively to have a true representation of data (Table 1). However, the response rate of survey forms was 97%.

**Table 1:** Demographic information and frequency of physical activity of participants.

Variables		***f***	***p***	***M***	***SD***
**Age**					
21 years		87	44 %	21.74	0.837
22 years		85	43 %		
23 years		20	10%		
24 years		2	1 %		
25 years		4	2%		
**Department & Tracks**					
*Rehabilitation Sciences*		64	31%		
	Physiotherapy	34	17%		
	Occupational Therapy	30	14%		
*Health Sciences*		75	37%		
	Epidemiology	19	10%		
	Health Education	21	10%		
	Clinical Nutrition	29	14%		
	Clinical Psychology	6	3%		
*Radiological Sciences*		31	15%		
	Diagnostic Radiation	13	6%		
	Ultrasound Therapy	17	8%		
	Radiology Therapy	1	1%		
*Communication Sciences*		34	17%		
	Speech & Language pathology	18	9%		
	Audiology and Balance	16	8%		
**Frequency of physical activity**					
Never		0	0	4.34	0.820
1-3 times a month		6	3%		
Once a week		26	13%		
Twice in a week		61	31%		
3-4 times a week		105	53%		
**Exercising last year**					
No, never exercised		0	0	3.47	0.666
yes, with longer interruptions		19	10%		
yes, with short interruptions		66	33%		
yes, without interruption		113	57%		

Note: *f* = frequency; *p* = percentage; *M* = mean; *SD* = Standard deviation.

Inclusion/exclusion criteria: The study was based on the perceived wellness of the students based on their physical activity engagement. The age range was 21–25 years (M±SD: 21.74 ± 0.837). There were a total of 255 students enrolled in final year BS programs at the CHRS. Students other than the final year and not meeting the age criteria were excluded. One of the Programs from the Rehabilitation department is Masters-degree program was excluded to avoid the maturity factor. Moreover, the Nuclear Medicine Technology program from the Radiology department was also excluded due to not offering final year levels for students. Therefore, the sample was collected from 11 programs.

### 2.1 Measures

i. *Demographic information sheet:* A personal information questionnaire prepared by the researchers was used to determine the age, department, track, and educational level.
ii. *Physical activity rating scale:* Physical activity rating scale was developed by Lippke and Ziegelmann (2006). It measures two main aspects of physical exercise. Fist aspect is frequency, and the second is constancy. The item of the frequency of exercise can be responded to on the 5-point Likert scale. Exercise consistency can be responded on a 4-point Likert scale. The current study found the Cronbach alpha of .79 for the physical activity rating scale.
iii. *Perceived wellness scale*: Perceived wellness scale (Adam et al., 1998) was designed to measure the wellness perception of individuals. It consists of 36 items (Adams et al., 1998). Overall, there are six dimensions, measures psychological wellness, social wellness, physical wellness, spiritual wellness, intellectual wellness, and emotional wellness. All the items can be rated on a 6-point Likert scale ranging from 1 (very strongly disagree) to 6 (very strongly agree). The score ranges from 36 to 216, and a higher score indicates a better perception of wellness. Scoring of dimensions can be summed and then divided by a number of items in dimensions (Adams et al., 1998). Various studies have reported the good validity and acceptable range of reliability of the measure (Adams et al., 1998; Rothmann and Ekkerd, 2007; Kaveh et al., 2016). The scale has been translated into Setswana and Persian languages and was found yielding valid results (Rothmann and Ekkerd, 2007; Kaveh et al., 2016). Perceived wellness scale was administered for health students in the English language as a standardized scale was not available in the Arabic language. Moreover, the targeted population was comfortable with English language comprehension. The current study found Cronbach alpha of .71 for wellness scale.

Ethical consideration: Detailed proposal of the study was submitted to the Institutional Review Board (IRB) of Princess Nourah bint Abdulrahman University before initiating the study, and permission was sought (IRB Log number: 18–0181). Consent forms, explaining the purpose of study, privacy, the confidentiality of data, indicating the role and responsibilities of the researcher, were provided to the subjects. After screening through demographic information, participants were given standardised scales. Data were collected by using individual administration of survey forms.

Statistical analysis: Collected data were analysed by using SPSS (V. 24). Descriptive Statistics of measures of central tendency and dispersion were calculated. Pearson product-moment correlation and multiple linear regression analysis were computed to find out the relationship and predictive association of physical activity and perceived wellness scores. Further, one-way ANOVA was conducted to measure the differences in wellness dimensions between the scores of students from four departments.

## 3 Results

Overall, 198 individuals participated in the survey. The response rate was 97%, with a refusal rate of 3%. Results in Table 1 is showing that the highest percentage of students were at the age of 22 years (43%). Students from four departments, including Rehabilitation (31%), Health (37%), Radiology (15%) and Communication Sciences (17%) responded. The highest ratio of the frequency of physical activity of 105 respondents (53%) replied that they are executing exercise 3–4 times a week (M±SD:4.34 ± 0.820). Whereas, no one responded in the category of never exercised. On the rating scale of exercising last year, 133 (57%) respondents replied as yes (M±SD:3.47 ± 0.666), without interruption, they were engaged in exercise.

Table 2 presented the Pearson correlation matrix is having a liner relationship between overall wellness and physical activity (frequency: = .330, p = .000; Exercising last year: r = .209, p = .003) and the different dimensions of wellness (psychological: r = .386, p = .000; emotional: r = .184, p = .010; social: r = .572, p = .000; physical: r = .653, p = .000; spiritual: r = .353, p = .000 and intellectual wellness: r = .350, p = .000) among students of CHRS. From the results it can be seen that physical wellness dimension was significantly correlated with all the factors including physical activity (frequency: = .444, p = .000; Exercising last year: r = .408, p = .000) social, intellectual and overall wellness (p < .01); and psychological, emotional and spiritual wellness (p < .05).

**Table 2:** Relationship between physical activity and dimensions of wellness scale.

Variables	Exercising last year	Psychological wellness	Emotional wellness	Social wellness	Physical wellness	Spiritual wellness	Intellectual wellness	Overall Wellness
Frequency of physical activity	.467**	.014	.057	.229**	.444**	.007	.032	.330**
Exercising last year		.243**	.095	.079	.408**	.103	.260**	.209**
Psychological wellness			.175*	.081	.152*	.134	.147*	.386**
Emotional wellness				.116	.171*	.233**	.238**	.184**
Social wellness					.399**	.216**	.029	.572**
Physical wellness						.177*	.268**	.653**
Spiritual wellness							.098	.353**
Intellectual wellness								.350**

**p < .01, *p < .05.

Table 3 shows the multiple linear regression analysis that was used to predict wellness from two variables of the frequency of physical activity and experience of exercise last year among the CHRS students. Both predictors, frequency of the physical activity, and exercising last year were found significantly predicting wellness (p < .01). The regression model was able to account for 11% of the variance by frequency of physical activity (R^2^ = .113, F (2, 195) = 12.371, p < .01) on the variable of wellness perception. Therefore, the first hypothesis of physical activity and perceived wellness association among students of Health and Rehabilitation Sciences has been accepted.

**Table 3:** Predictive association of physical activity and exercising last year with overall wellness perception.

Variables	Exercising last year	Overall Wellness	R^2^	F	SE	df	p
Frequency of physical activity	.467**	.330**	.113	12.371	5.388	2	.000**
Exercising last year		.209**					

Note: **p < .01.

Table 4 One-way ANOVA showed second hypothesis was also accepted that by comparing department wise scores of students on dimensions of wellness there are significant difference on psychological wellness (F (3,197) = 25.377, P = .000), Emotional wellness (F (3,197) = 4.653, P = .004), social wellness (F (3,197) = 22.278, P = .000), physical wellness (F (3,197) = 102.087, P = .000), spiritual wellness (F (3,197) = 6.586, P = .001), intellectual wellness (F (3,197) = 10.587, P = .000) and overall wellness (F (3,197) = 38.644, P = .000).

**Table 4:** Comparisons of departments on wellness dimensions.

Dimensions of wellness	Departments	N	M	SD	F	p
Psychological wellness	Rehabilitation Sciences	62	30.03	1.873	25.377	.000
	Health Sciences	73	32.73	2.323		
	Radiological Sciences	30	29.90	2.412		
	Communication Sciences	33	29.45	2.623		
Emotional wellness	Rehabilitation Sciences	62	30.82	1.833	4.653	.004
	Health Sciences	73	31.85	2.623		
	Radiological Sciences	30	31.50	2.113		
	Communication Sciences	33	32.39	1.171		
Social wellness	Rehabilitation Sciences	62	34.24	1.112	22.278	.000
	Health Sciences	73	33.66	1.204		
	Radiological Sciences	30	31.83	2.276		
	Communication Sciences	33	32.27	2.125		
Physical wellness	Rehabilitation Sciences	62	35.29	1.030	102.087	.000
	Health Sciences	73	30.58	2.134		
	Radiological Sciences	30	30.57	2.029		
	Communication Sciences	33	29.85	2.108		
Spiritual wellness	Rehabilitation Sciences	62	34.44	1.223	6.586	.000
	Health Sciences	73	33.53	2.028		
	Radiological Sciences	30	32.90	1.709		
	Communication Sciences	33	33.42	1.678		
Intellectual wellness	Rehabilitation Sciences	62	33.53	1.817	10.587	.000
	Health Sciences	73	32.63	2.058		
	Radiological Sciences	30	33.87	1.137		
	Communication Sciences	33	31.64	2.028		
Overall Wellness	Rehabilitation Sciences	62	198.35	3.918	38.644	.000
	Health Sciences	73	194.97	4.082		
	Radiological Sciences	30	190.57	5.469		
	Communication Sciences	33	189.03	5.559		

Note: N = 198; M = mean; SD = standard Deviation; p < .05.

**Figure 1:**
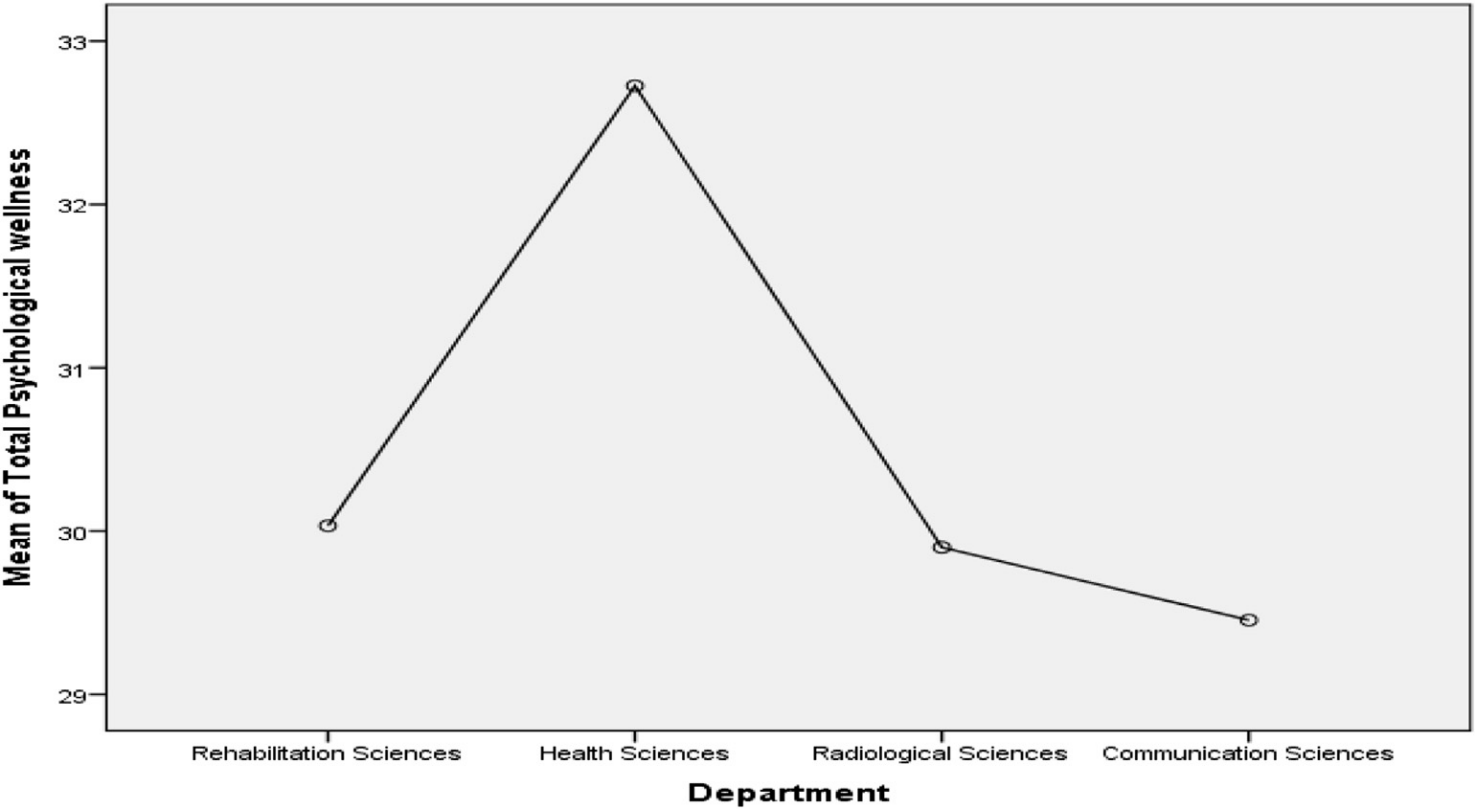
Mean Plot of Scheffé posthoc comparisons for Psychological Wellness.

**Figure 2:**
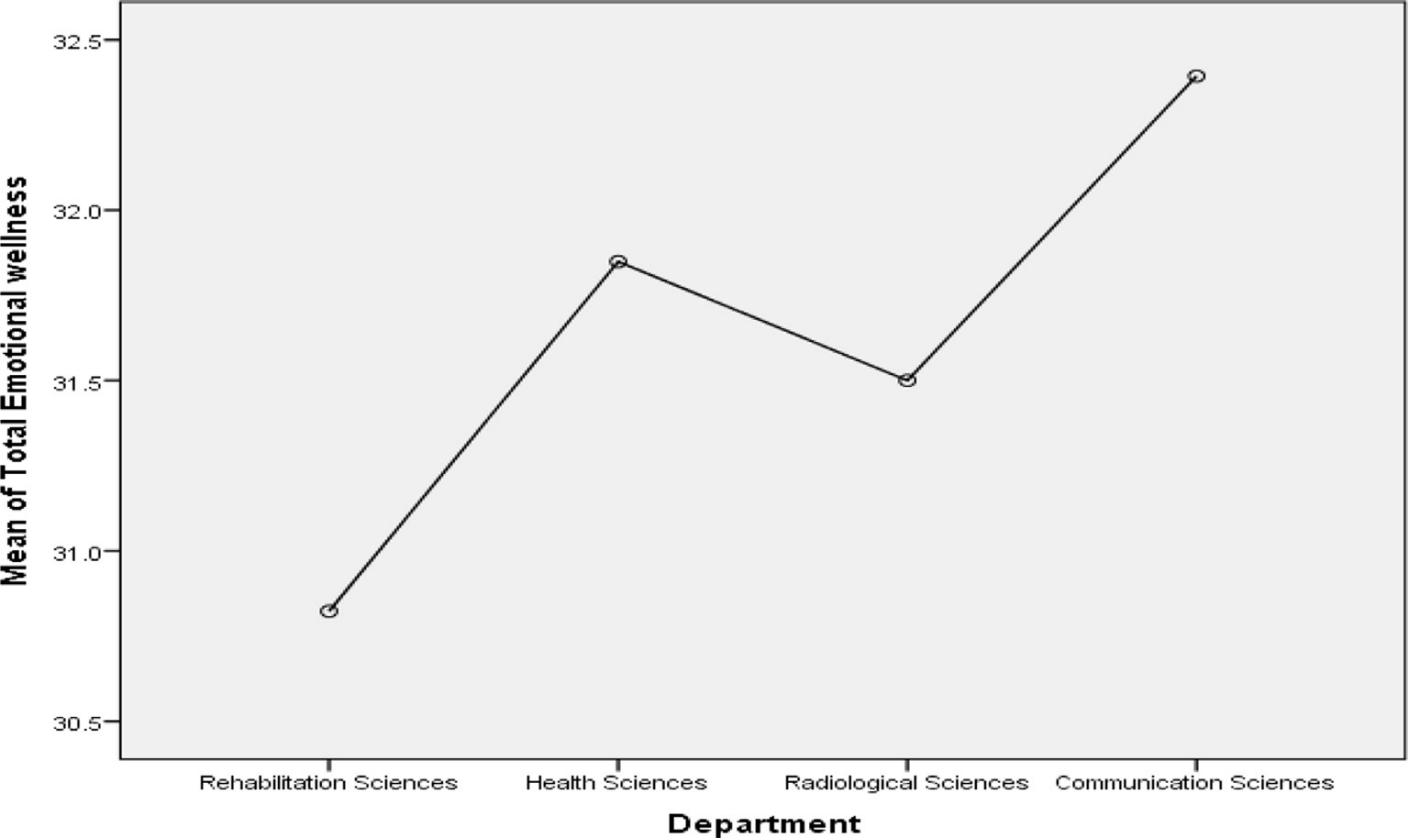
Mean Plot of Scheffé posthoc comparisons for Emotional Wellness.

**Figure 3:**
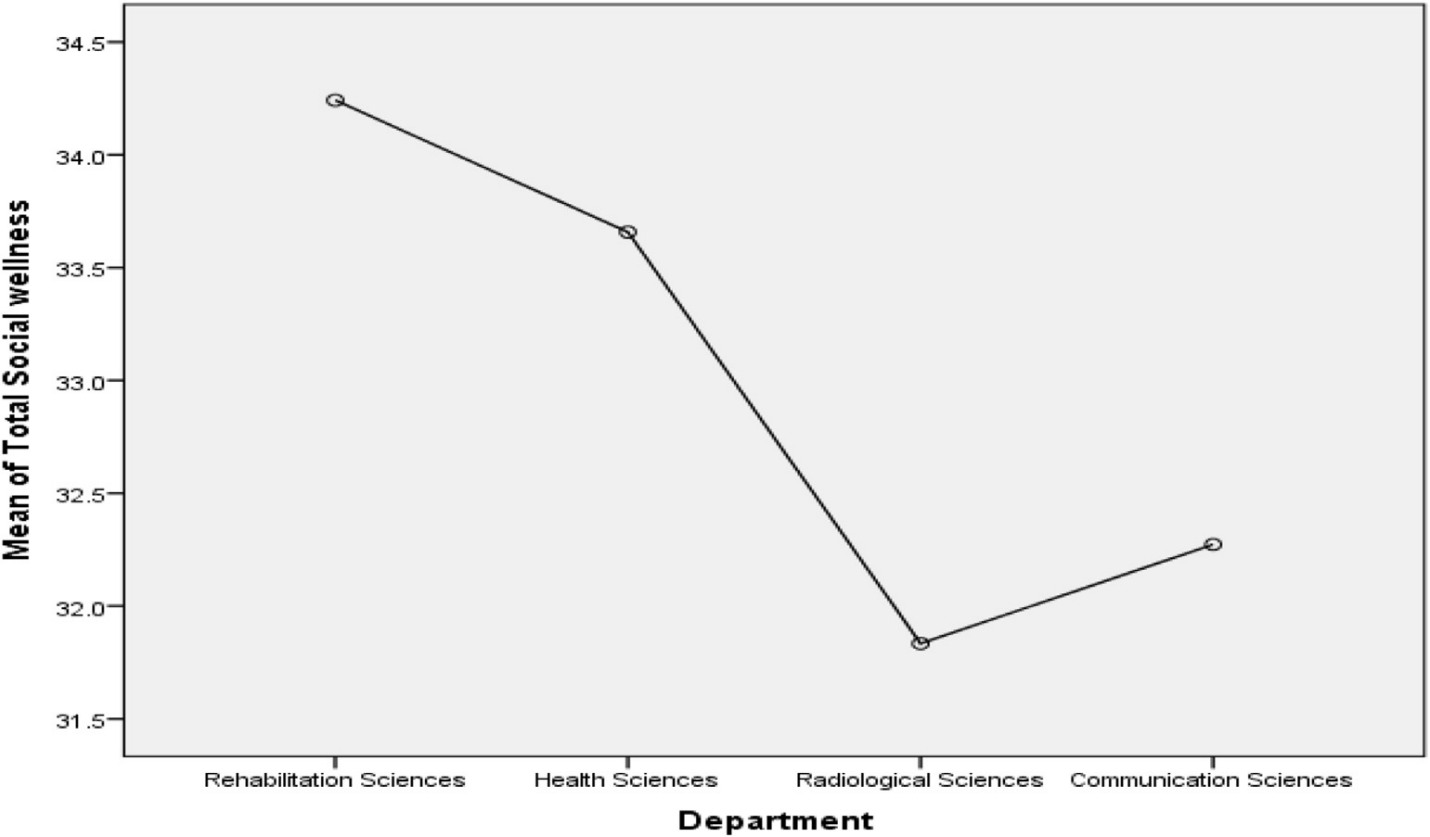
Mean Plot of Scheffé posthoc comparisons for Social Wellness.

**Figure 4:**
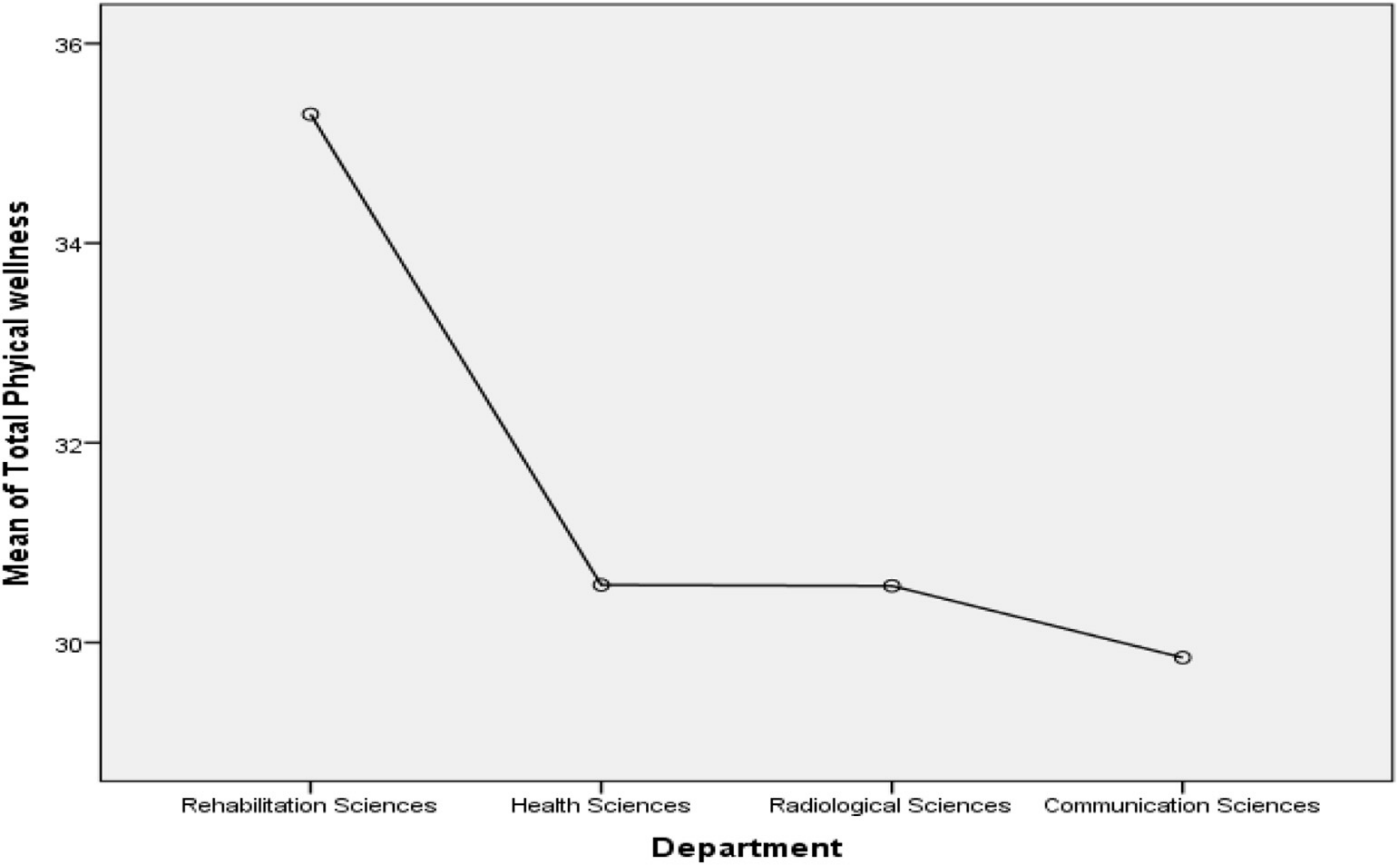
Mean Plot of Scheffé posthoc comparisons for Physical Wellness.

**Figure 5:**
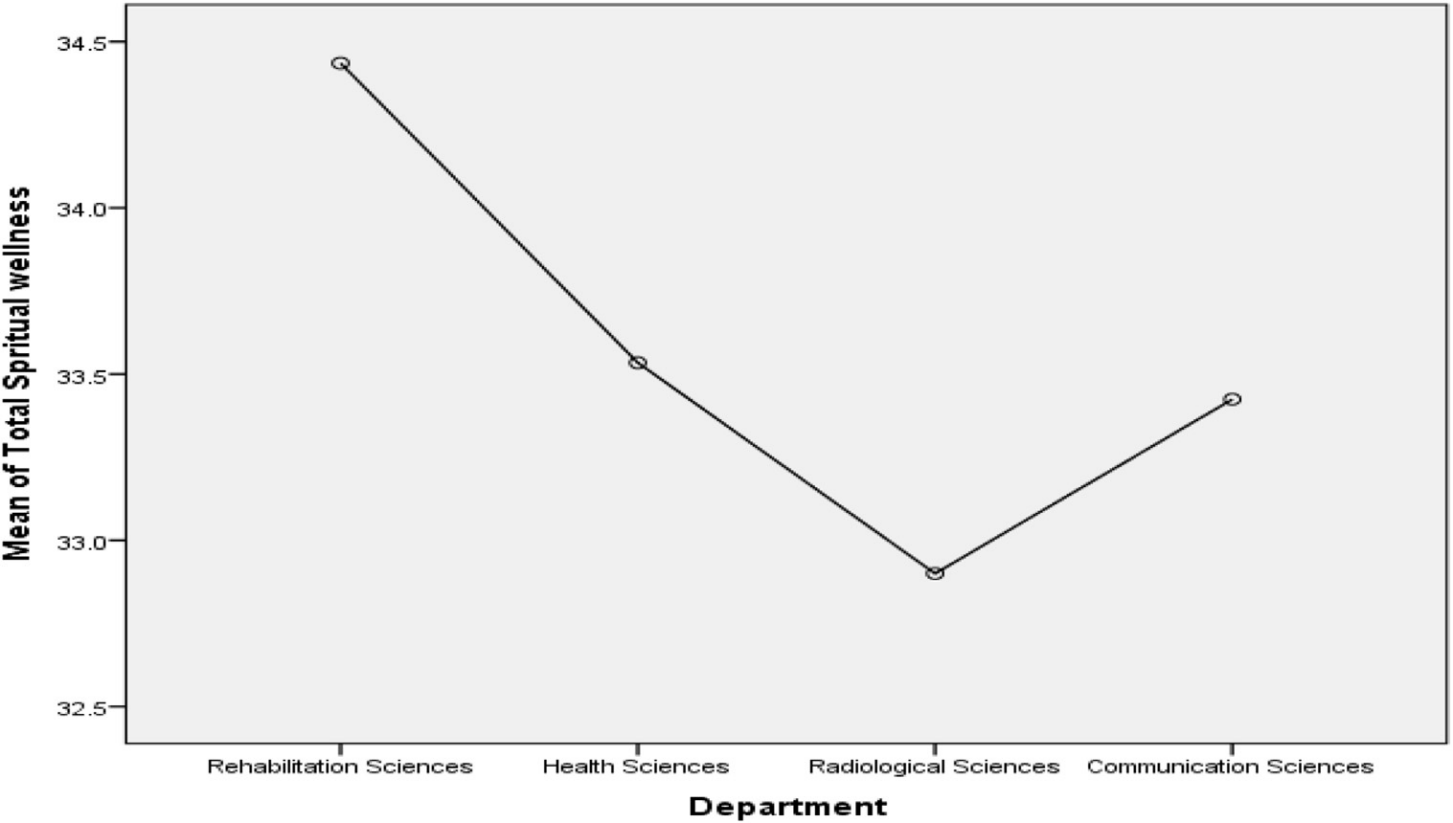
Mean Plot of Scheffé posthoc comparisons for Spiritual Wellness.

**Figure 6:**
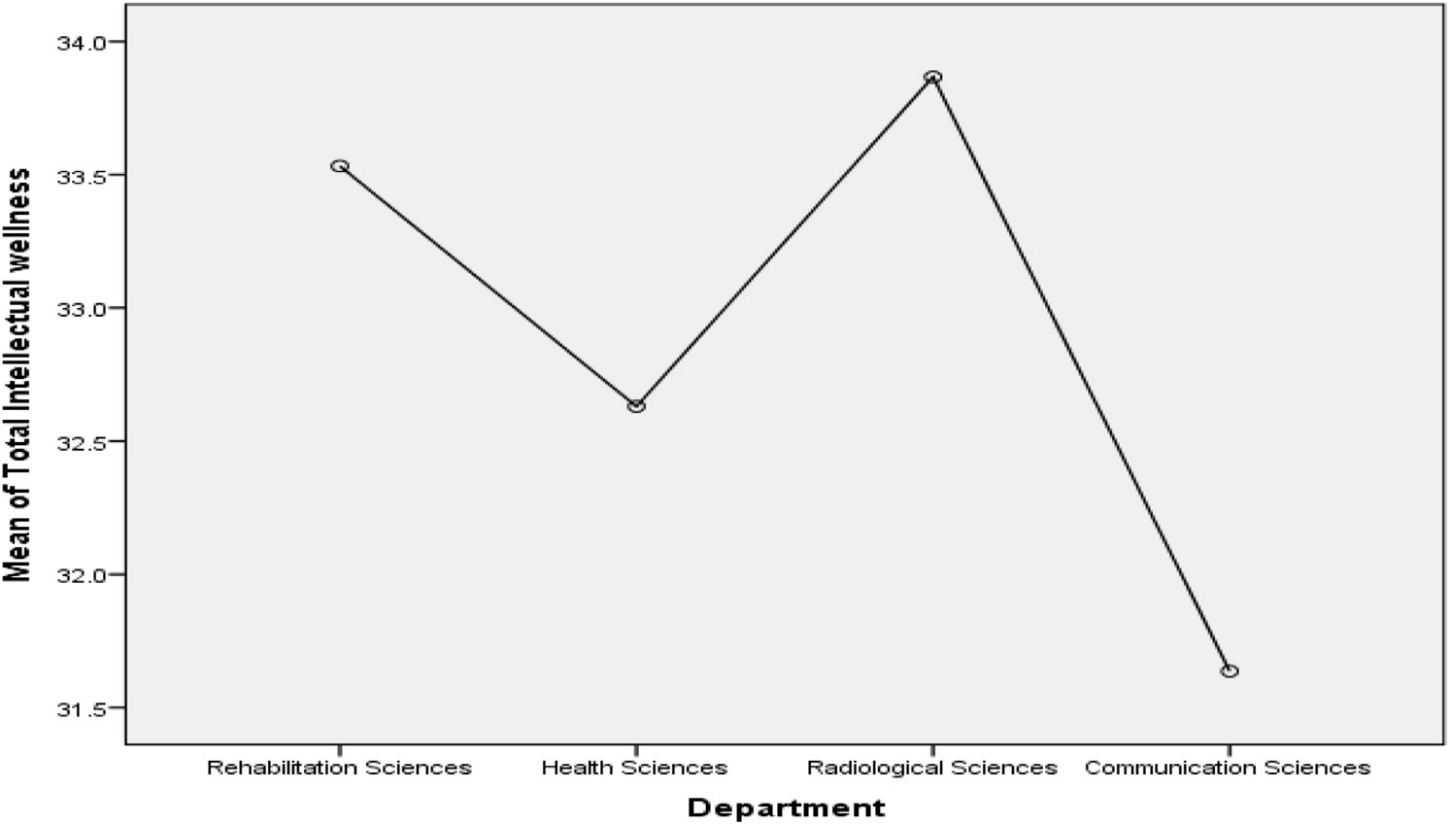
Mean Plot of Scheffé posthoc comparisons for Intellectual Wellness.

**Figure 7:**
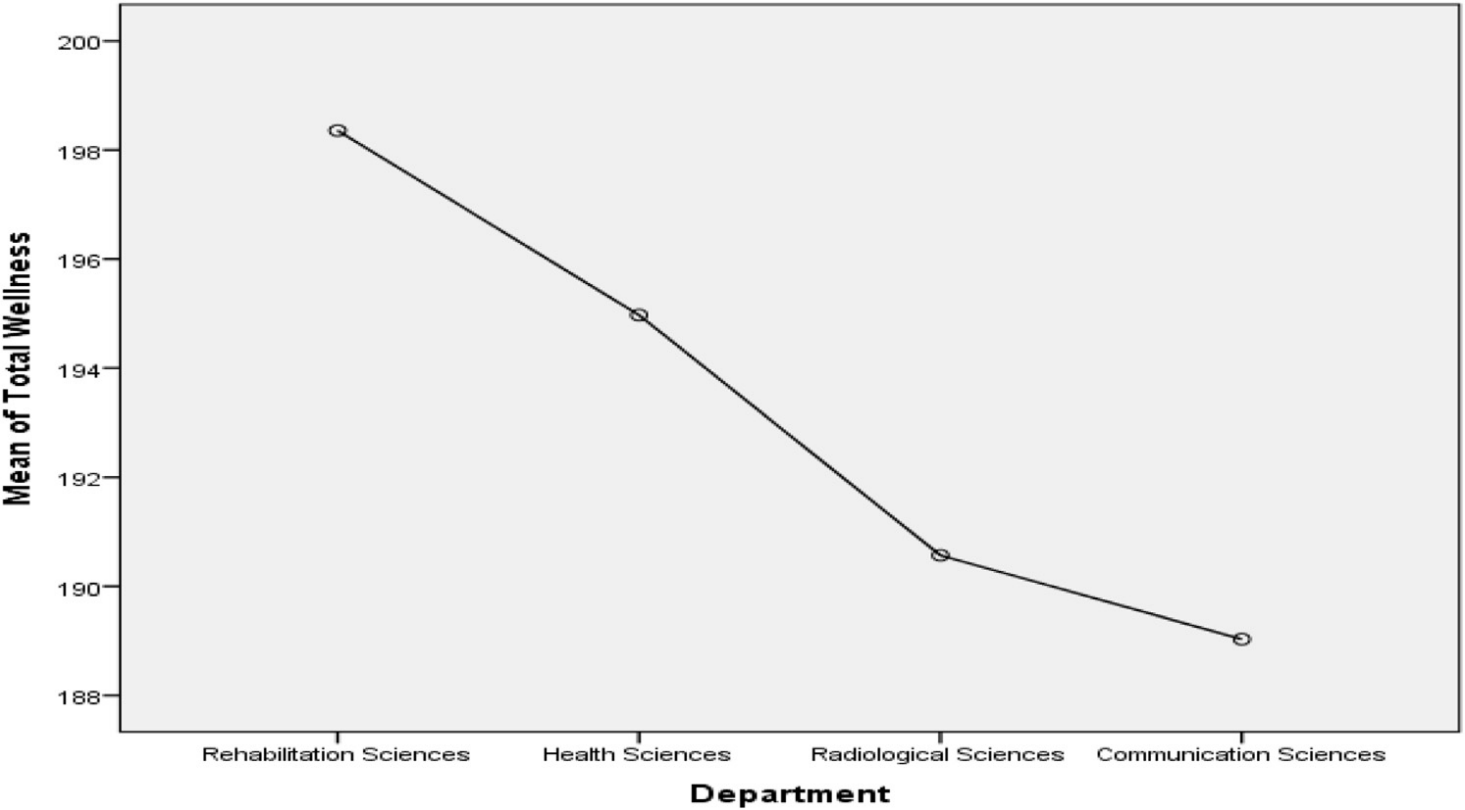
Mean Plot of Scheffé posthoc comparisons for Overall Wellness.

Scheffe post-test comparison (Figure 1) indicated that Health sciences students score higher mean on psychological wellness (M = 32.73) as compare to Rehabilitation (M = 30.03), Radiology (M = 29.90) and Communication Sciences (M = 29.45) departments, p < .05. On emotional wellness (Figure 2), two departments score differently. Communication Sciences students (M = 32.39) score highest on emotional wellness, whereas, Rehabilitation sciences students (M = 30.82) score lowest as compare to Health sciences (M = 31.85) and Radiological sciences (M = 31.50) on emotional wellness, p < .05. Rehabilitation (M = 34.24) and Health (M = 33.66) sciences students score higher mean on social wellness (Figure 3) than other departments. It can be seen in Figure 4 that the Rehabilitation sciences scored higher on the physical wellness (M = 35.29) domain as compared to all other departments. On spiritual wellness (Figure 5), Rehabilitation Sciences score (M = 34.44) higher and Radiological Sciences (M = 32.90) score prominently lower. On intellectual wellness (Figure 6), Radiological Sciences scored higher mean (M = 33.87), and Communication Sciences scored lower mean (M = 31.64) as compared to others. On overall wellness (Figure 7) mean score Rehabilitation scored higher (M = 198.35) than Health Science (M = 194.97), whereas, Radiological Sciences and Communication Sciences scored as lower (M = 190.57 & M = 189.03) than others.

## 4 Discussion

The general awareness of the concept of wellness makes the health sciences students value the importance of the relationship that exists between the sound nourishment and functioning of the body. Moreover, the wellness perception motivates the students toward engagement in physical exercises. The medical students require several dimensions of wellness perception in order to enhance their lifestyles in a healthy manner and aid in the achievement of general wellness (Rehman et al., 2013).

Physical activity has been found to contribute immensely to the general well-being of an individual (Zayed et al., 2018). The benefits associated with the physical exercise contributes toward reducing stress level, development of stronger and healthier muscles, and to enhance the immune system. According to the World Health Organisation report, individuals who engage in physical activities and maintaining a proper dietary plan, most frequently, have increased life expectancy, increased levels of energy, and less susceptible to diseases in their daily routine. In the previous study, it was mentioned that for optimal physical wellness an individual (WHO, 2018) ought to know how to identify illness symptoms, go for frequent medical check-ups, to maintain proper dietary practices, and to refrain from drug abuse (Fatima et al., 2013). Previous studies have observed that the medical students were found to have a high level of physical activity because of their university modules involving sports as well as their free-time physical activities (Mikolajczyk et al., 2008; Lobelo et al., 2009). The results of the current study were in line to accept the hypothesis that the health and rehabilitation students’ scores were found highly associated with physical activity and overall wellness. It is because they are more knowledgeable about the benefits that are acquired from engaging in physical exercises (Mikolajczyk et al., 2008).

Research has found that the wellness of human beings comprises several dimensions that must be attained to fully achieve wellness, including spiritual wellness, emotional wellness, psychological wellness, intellectual wellness, social wellness, physical wellness, and even financial wellness (WHO, 2002). In the current study, findings are indicating that statistically, it has been proved that health sciences students have scored high on all the dimensions of wellness and overall wellness as well. It further leads toward the optimistic view that learning and practicing wellness dimensions are promoting health perception among Saudi female health students (Bandura, 1986; Melchert, 2011). Despite the existence of these dimensions, one might be surprised by how few individuals know about them properly. The second hypothesis was also accepted that department wise there was a difference in various dimensions of wellness. Across the departments of the CHRS, the pattern developed was that the Rehabilitation students showed higher awareness of physical, social and spiritual wellness, and Health sciences students showed higher awareness of psychological and social wellness. Communication sciences students showed awareness for emotional and Radiological sciences performed higher on intellectual wellness. This variation might be explained due to their interest in their disciplines. Rehabilitation Sciences department consist of programs of Physiotherapy and Occupational therapy. Both programs focused on physical activities, healthy interaction toward patients and to give hope and an optimistic approach to their clienteles (Verhagen and Engbers, 2009; American Physical Therapy Association (APTA), 2014). Health Sciences department is consisting of Epidemiology, Health education, Clinical Nutrition, and Clinical Psychology. It is evident from findings that all of these four programs are preparing their students to focus on psychological and social wellness. Without these proper psychological understanding, students cannot develop a good rapport with their clients. Furthermore, for the adherence of treatment and psychoeducation, excellent therapeutic skills count a lot (Casañas et al., 2015). Simultaneously, students of Communication has to deal with children population suffering from disabilities, so they require more emotional wellness and stability, whereas, Radiology students concerned toward the suitable selection of modality and diagnostic tools thus more focus on intellectual wellness.

Concerning other dimensions of wellness, the Health and Rehabilitation students scored relatively low on specific domains. It may be due to the rigorous training in their specific discipline. Previous studies have established that medical training exposed more psychosocial stress among female students than other types of education (Abdulghani et al., 2011). Therefore, physical activity can reduce the level of stress and promote wellbeing. Various departments and programs of the CHRS can implement the findings to enhance the achievement of overall wellness. In future studies, other aspects can be explored to measure the relationship between nutrition, lifestyles, and training with physical activity among Saudi male and female health sciences students.

## 5 Conclusion

In conclusion, the students from health and rehabilitation sciences showed an exceptional awareness of the perception of all the six dimensions of wellness, including psychological, social, emotional, spiritual, physical, intellectual wellness, and overall wellness as well. Physical activity was found associated and can predict wellness among health students. What was established is that the health and rehabilitation students were found different in various dimensions of wellness. Moreover, overall, students are responding to their wellness and practicing physical activities regularly.

## Declarations

### Author contribution statement

U. Zaidi: Conceived and designed the experiments; Performed the experiments; Analyzed and interpreted the data; Contributed reagents, materials, analysis tools or data; Wrote the paper.

### Funding statement

This research was funded by the Deanship of Scientific Research at Princess Nourah Bint Abdulrahman University, Saudi Arabia through the Fast-track Research Funding Program.

### Competing interest statement

The authors declare no conflict of interest.

### Additional information

No additional information is available for this paper.
